# Game-based learning environments affect frontal brain activity

**DOI:** 10.1371/journal.pone.0242573

**Published:** 2020-11-19

**Authors:** Silvia Erika Kober, Guilherme Wood, Kristian Kiili, Korbinian Moeller, Manuel Ninaus

**Affiliations:** 1 Institute of Psychology, University of Graz, Graz, Austria; 2 BioTechMed-Graz, Graz, Austria; 3 Faculty of Education and Culture, Tampere University, Tampere, Finland; 4 Centre for Mathematical Cognition, School of Science, Loughborough University, Loughborough, United Kingdom; 5 Leibniz-Institut für Wissensmedien, Tübingen, Germany; 6 LEAD Graduate School & Research Network, Eberhard-Karls University Tübingen, Tübingen, Germany; NASA Johnson Space Center, UNITED STATES

## Abstract

Inclusion of game elements in learning environments to increase motivation and learning outcome is becoming increasingly popular. However, underlying mechanisms of game-based learning have not been studied sufficiently yet. In the present study, we investigated effects of game-based learning environments on a neurofunctional level. In particular, 59 healthy adults completed a game-based version (including game elements such as a narrative and virtual incentives) as well as a non-game-based version of a number line estimation task, to improve fractional knowledge, while their brain activity was monitored using near-infrared spectroscopy. Behavioral performance was comparable across the two versions, although there was a tendency that less errors were made in the game-based version. However, subjective user experience differed significantly between versions. Participants rated the game-based version as more attractive, novel, and stimulating but less efficient than the non-game-based version. Additionally, positive affect was reported to be higher while engaging in the game-based as compared to the non-game-based task version. Corroborating these user reports, we identified increased brain activation in areas associated with emotion and reward processing while playing the game-based version, which might be driven by rewarding elements of the game-based version. Moreover, frontal areas associated with attention were also more activated in the game-based version of the task. Hence, we observed converging evidence on a user experience and neurofunctional level indicating that the game-based version was more rewarding as well as emotionally and attentionally engaging. These results underscore the potential of game-based learning environments to promote more efficient learning by means of attention and reward up-tuning.

## Introduction

In recent years, game elements have been incorporated into learning tasks in education with increasing frequency [1–3]. Game-based learning environments are assumed to increase interest, motivation, adherence to instructions, and, consequently, the learning outcome [2–4].

Game elements may include game mechanics (e.g., core activities repeated by the learner throughout the game), visual aesthetics, narrative of a game (storyline), incentives such as scores (points), stars, badges, trophies, power-ups, or any other rewards, or sounds [5]. Generally, games or game elements are assumed to be rewarding [2, 6–11]. For instance, King et al. (2011) [12] found that game elements such as incentives (earning points) were rated as the most enjoyable and important aspects of video game playing. Moreover, providing concise, real-time feedback of actual performance by means of game elements such as rewards was found to lead to stronger engagement of the learner [5, 9, 13, 14]. As such, there is already evidence that game-based learning environments lead to improved learning outcomes compared to conventional educational approaches [3, 4]. However, there is also consensus that more studies in terms of large randomized control trials are necessary to better understand underlying working mechanisms in game-based learning tasks and to draw a meaningful conclusion [2, 3]. Although the number of studies investigating behavioral effects of game-based learning is increasing, effects of game-based learning environments on brain activation patterns, which are associated with learning processes, are investigated only scarcely still.

In this context, we investigated neuronal correlates of playing a game-based and a non-game-based version of a number line estimation task which requires participants to indicate the correct location of a given target number on a number line of which only start and endpoint are given. Importantly, this task was found to assess and train number magnitude understanding [15, 16]. The game-based and non-game-based number line estimation solutions of the current study were developed with the *Semideus* research engine which allows for customization of game elements and was originally developed to improve conceptual fractional knowledge in children [17]. In both versions, participants performed the number line estimation task, in which they had to indicate the position of fractions (e.g., 3/7) on a horizontal line ranging from 0 to 1. In contrast to the non-game-based version, the game-based version included game elements such as a narrative and virtual incentives. Positive/negative feedback was presented in both task versions.

Behavioral studies showed that game-based versions of the number line estimation task can be used to effectively train and assess fractional knowledge in children [17–20]. To examine whether the inclusion of game elements affects brain activation patterns when performing the number line estimation task, we used near-infrared spectroscopy (NIRS). NIRS measures changes in the hemodynamic response (relative concentration changes of oxygenated and deoxygenated hemoglobin in the outer layer of the cortex; oxy- and deoxy-Hb) of the brain. Generally, neuronal activation in specific brain areas leads to a localized vascular response that causes an influx of oxygen-rich blood to the active area and its surrounding tissue. Consequently, this leads to an increase in oxy-Hb and a decrease in deoxy-Hb in active brain regions [21]. NIRS is a non-invasive, portable, easy-to-use, and inexpensive neurofunctional method that does not restrict participants’ movements or natural behavior while performing a task as compared to other methods (e.g. the electroencephalogram -EEG- or functional magnetic resonance imaging -fMRI-) [22].

The majority of studies investigating neuronal correlates of gaming used EEG or fMRI. According to a review article by Palaus et al. (2017), 8 studies used NIRS to investigate neuronal correlates of gaming. In these NIRS studies, different types of games were used such as action games, ego-shooters, puzzle games, strategy games, rule learning games, or racing games [10]. Therefore, reported changes in brain activity due to gaming were rather heterogeneous. Some studies reported a global decrease of oxy-Hb over frontal brain areas during gaming [23–27]. However, differences in task complexity, cognitive load, expertise, or subjective user experience during playing may have added to these different brain activation patterns [28–32].

Effects of game-based learning environments on brain activation patterns have hardly been investigated so far. In a pilot study, Baker et al. (2015) investigated neuronal correlates during playing a computer-based fraction learning game called *Refraction* using NIRS. Refraction requires the player to combine math (i.e., fractions) and spatial (i.e., screen navigation) skills to complete a series of game stages successfully. Beside the combined math and spatial tasks, the authors also added two control conditions in which participants either performed only math or spatial tasks. They found stronger activation within parietal and prefrontal brain regions when participants performed the combined tasks in the game Refraction compared to solely spatial activities or math tasks. They interpreted the observed increased activation over prefrontal areas when playing Refraction as a sign of increased working memory load and attentional demands when playing the game Refraction [33].

In the present study, our primary aim was to investigate a larger sample of adults and directly compare brain activation patterns associated with playing a game-based (including game elements) and a content-wise equivalent non-game-based version [17]. Game elements are assumed to increase engagement in the task, positive affect, motivation, and consequently the learning outcome [5, 10, 11, 14]. In particular, there is empirical evidence that such game elements can activate the reward system in the brain [6, 10, 34–38]. On a general level, previous fMRI studies showed that reward and emotion processing can increase prefrontal brain activation, which was associated with working memory and attention [36, 39–45] [for general reviews on prefrontal function and location see 46, 47]. Furthermore, previous fMRI studies indicated that spatial-numerical tasks in general [48–50] and the number line estimation task in particular [51], lead to neuronal activation in a fronto-parietal brain network. Therefore, we also focused specifically on changes in brain activation patterns over prefrontal areas during gaming. As in the current study, task content and complexity were held constant across the game-based and the non-game-based version, differences in brain activation patterns between versions should be related to the incorporation of game elements. We expected the game elements to be rewarding and emotionally engaging [7, 8, 14] and thus to lead to stronger prefrontal brain activation compared to the non-game-based version.

Beside our main research question concerning effects of a game-based learning environment on brain activation patterns, we also investigated behavioral effects as secondary research question. Concerning task performance, there are heterogeneous results reported in the literature. Some studies reported beneficial effects of game-based tasks on behavioral performance, other studies argued that game elements might induce cognitive overload and distract learners from the actual task [3–5, 9, 11, 52–57]. Therefore, we also analyzed possible differences in task performance between the two versions.

In a tertiary and more exploratory research question, we focused on subjective user experience including flow and affective state, when performing both versions of the task. Prior studies showed that a game-based version of a task can lead to a stronger flow experience and higher user experience ratings than a non-game-based version of the same task [14, 58–61]. Flow is defined as a positive emotional state of optimal performance [62]. Generally, flow experience seems positively related with learning outcomes and playing performance in game-based learning approaches [58–60, 63, 64] and was often associated with positive user experience [58, 65]. However, there are also studies that found no differences in flow experince between game-based and non-game-based versions of the same task [9]. Therefore, we also investigated the subjective user experience, flow, and affective state while playing both task versions in the present study.

## Methods

### Participants

Fifty-nine right-handed healthy adults (29 female, 30 male, mean age = 22.85 yrs., *SD* = 3.60; all were Caucasian) took part in this study. Fifty-one were students, eight were employees (lawyers, medical doctors, software engineers). They had normal or corrected-to-normal vision. Participants were recruited via fliers and advertisements, personal recruitment, as well as via mailing lists of the University of Graz. They did not get any financial compensation. Psychology students got a partial course credit. Participants gave written informed consent prior to testing. The study was approved by the Ethics Committee of the University of Graz, Austria (reference number GZ. 39/86/63 ex 2017/18) and is in accordance with the ethical standards of the Declaration of Helsinki.

### Task–game-based vs. non-game-based task

**Fig 1:**
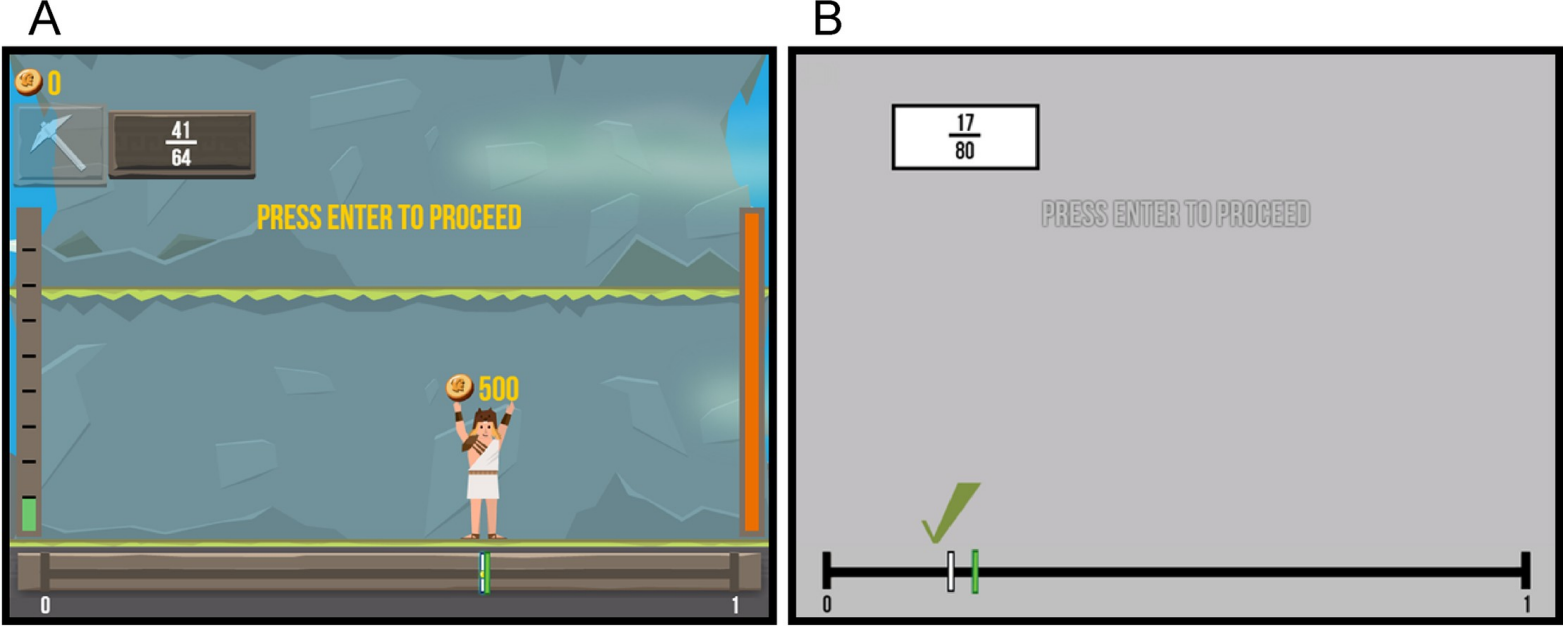
Examples scenes of the game-based and non-game-based task version. Exemplary screenshots of the game-based version (A) and the non-game-based version (B) of the number line estimation task when receiving positive feedback after correct estimation.

All participants performed a game-based and a non-game-based version of a number line estimation task. Half of the participants started with the game-based version, the other half with the non-game-based version. In the number line estimation task, participants had to locate a given target fraction (e.g., 3/7) on a number line ranging from 0 to 1. In each version (game-based vs. non-game-based) 48 fractions involving single- (e.g., 3/7) and double-digit (e.g., 19/25) numerators and denominators were presented. The game-based version of the number line estimation task (see Fig 1A) was realized with the Semideus research game engine, which allows for easy inclusion of several game elements [17, 19]. The non-game-based version of the number line estimation consisted of a stripped version of the task without any game elements (Fig 1B) but maintained positive/negative feedback. It corresponds to the conventional number line estimation task [e.g., 15]. Both versions were comparable regarding task difficulty as they involved the same target fraction as well as number of levels (i.e., 6 levels, each including 8 tasks/fractions).

In both versions, a target fraction to-be-located on the number line was displayed in the upper left part of the screen. Participants could move a cursor (white bar in the non-game-based version) or the game character Semideus (in the game-based version) on the number line with the left and right arrow keys on a standard QWERTZ keyboard and confirm their estimates by pressing the space bar. Feedback was provided immediately after each estimation. In the non-game-based version, positive feedback was given by showing a green check mark above the cursor in case the target fraction was estimated accurately (i.e., estimated location no more than ±5% away from the correct location) and at the same time the absolute correct location was shown by a green marker on the number line. Negative feedback was given in the form of a red cross in case the estimated location was more than ±5% away from the correct location. The same negative feedback mechanic was shown when the estimation took too long (>10 seconds).

In the game-based version, positive feedback was provided through gestures of Semideus (e.g. lifting hands up and cheering) when estimations were correct (i.e., estimated location no more than ±5% away from the correct location). Additionally, participants earned virtual coins for accurate estimations (i.e., over 98% = 500 coins; 97%–98% = 300 coins; 96%–95% = 100 coins). For inaccurate estimates (i.e., estimates more than ±5% away from the correct location) the game character Semideus was struck by lightning and the player lost 10 units of virtual energy (displayed by an orange bar on the right side of the screen, see Fig 1A) on the first error in a trial, 5 units of energy for the second error on the same trial, and 2.5 units of energy for any further error on that same trial/item. The same negative feedback mechanic (loss of energy and lightning) was used when players took too long to make their estimation (i.e., pressing the space bar, >10 seconds). This time limit was visualized by a cloud getting darker with passing time and a numerical countdown within the cloud.

In general, the game-based version employed typical characteristics of games (Plass et al., 2015) such as narrative elements (story line: Semideus tries to find gold coins that a goblin has stolen from Zeus and hidden along the trails of Mount Olympus), appealing visual aesthetics, virtual incentives in the form of points and stars earned depending on the performance of participants, as well as positive/negative feedback. A comprehensive description of the game elements used in the game-based version and the rational of using them can be found in Ninaus et al. (2019) [14].

The number of estimation attempts on each task was not limited and participants had to estimate the location of the fraction correctly before they were allowed to proceed to the next task. After correct estimations, participants were asked to press the Enter-key to proceed to the next task.

In both versions, the absolute number of errors, mean estimation accuracy, and mean duration per task/fraction were used as dependent variables for statistical analysis.

### NIRS-recordings and -analysis

**Fig 2:**
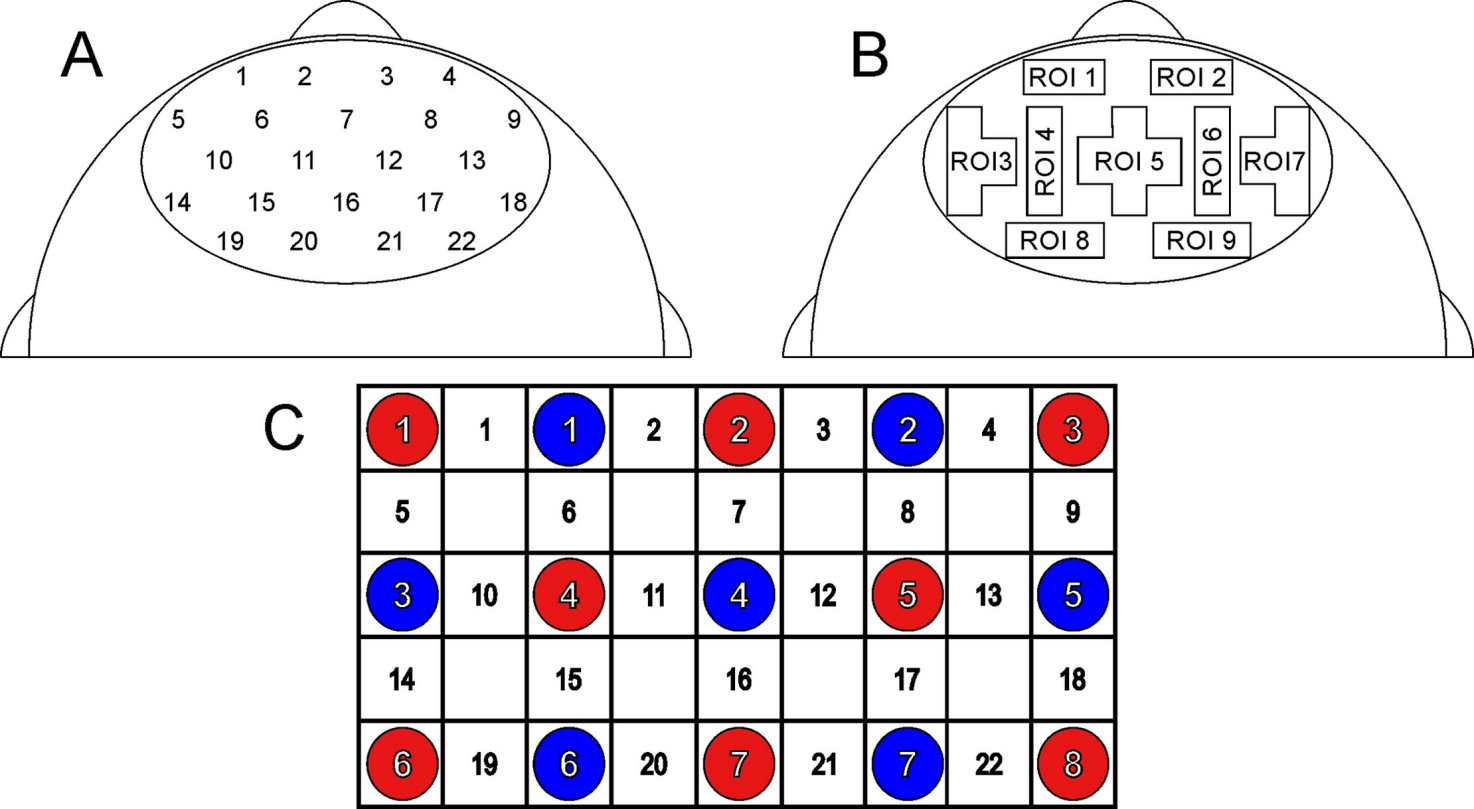
Position of the optode probe set on the head of participants. (A) Placement of the 22 NIRS channels on the forehead. (B) Positions of the nine regions of interests (ROIs). (C) Channel configuration and positions of the emitters (red circles) and detectors (blue circles) of the optode probe set (3x5). Numbers in the white rectangles represent the respective NIRS channel number.

To measure relative concentration changes of oxygenated (oxy-Hb) and deoxygenated hemoglobin (deoxy-Hb) over frontal areas during the game-based and non-game-based version of the line estimation task, measurements were performed on a continuous wave system (ETG-4000, Hitachi Medical Co., Japan) using two 3x5 optode probe sets (consisting of 7 photo-detectors and 8 light emitters) resulting in a total of 22 channels (see Fig 2). The ETG-4000 uses two different wavelengths (695±20 nm and 830±20 nm). Distance between optodes was 3 cm when mounted. Sampling rate of the NIRS system was set to 10 Hz. Channel configuration of the NIRS probe set is given in Fig 2. It was positioned over the forehead. In accordance with the international 10–20 placement system, we used Fz (emitter 7) as marker position to place the probe set. The 22 NIRS channels were merged to regions of interest (ROI) for statistical analysis (Fig 2B).

The MNI coordinates of the NIRS channels were determined using AtlasViewer [66]. In Table 1, the anatomic labels are listed for each NIRS channel.

**Table 1:** Anatomic labeling of NIRS channel positions (MNI coordinates) and merged regions of interests (ROI).

	Ch nr.	Emitter	Detector	Label name	Ch coord. MNI
**ROI1**	1	1	1	Orbitofrontal cortex left	-29 64–2
	2	2	1		-11 64 0
**ROI2**	3	2	2	Orbitofrontal cortex right	15 70–2
	4	3	2		26 51 2
**ROI3**	5	1	3	Middle frontal cortex left	-42 61 4
	10	4	3		-19 50 8
	14	6	3	Inferior frontal cortex left	-29 41 16
**ROI4**	6	4	1	Superior frontal cortex left	-14 52 4
	15	4	6		-10 45 20
**ROI5**	7	2	4	Medial superior frontal cortex	0 55 14
	11	4	4		-9 69 23
	12	5	4		12 51 18
	16	7	4		2 50 32
**ROI6**	8	5	2	Superior frontal cortex right	19 52 4
	17	5	7		21 57 28
**ROI7**	9	3	5	Middle frontal cortex right	37 48 2
	13	5	5		21 46 10
	18	8	5	Inferior frontal cortex right	37 40 18
**ROI8**	19	6	6	Middle frontal cortex left	-14 39 26
	20	7	6	Superior frontal cortex left	-8 55 44
**ROI9**	21	7	7	Superior frontal cortex right	13 55 48
	22	8	7	Middle frontal cortex right	22 41 30

Ch: Channel; MNI: Montreal Neurological Institute; ROI: Region of interest.

For offline analyses of the NIRS signal, we investigated changes in oxy-Hb and deoxy-Hb separately using the Homer2 NIRS Processing package [67] based on MATLAB (Mathworks, MA USA). The following NIRS signal processing steps were performed [68]:

The raw optical intensity data series were converted into changes in optical density (OD). Channels with very low optical intensity where discarded from the analysis using the function enPruneChannels. Then an automatic motion correction was applied using a wavelet transform method (function hmrMotionCorrectWavelet, iqr = 0.1) [69]. Additionally, artifacts, which were still in the signal were identified (function hmrMotionArtifact, signal change greater than std_threshold of 10 or amp_treshold of 5). The signal was filtered with a low-pass filter of 0.5 Hz. Then optical density data were converted into concentration changes (function hmrOD2Conc, ppf = 6.0 6.0). Marked artifacts were rejected (tRange -10 to 10 seconds).

The time courses of oxy-Hb and deoxy-Hb (hemodynamic response) of the remaining levels were averaged task-related separately for both versions (game-based and non-game-based version, average across 6 levels each) in each individual separately. Task-related concentration changes of oxy-Hb and deoxy-Hb were referred to a 5s baseline interval prior to level onset (seconds -5 to 0). For statistical analyses, oxy-Hb and deoxy-Hb was averaged for the time period during the task (0–50 s after level onset).

### Questionnaires

To assess subjective experience of participants, they had to fill in the Flow Short Scale (FKS) [70], the User Experience Questionnaire (UEQ) [71], and the German version of the Positive and Negative Affect Schedule (PANAS) [72] once after the game-based and the non-game-based version of the number line estimation task each.

The Flow Short Scale (FKS) [70] assesses flow experience using 10 items. Six of the items measure “fluency of performance” (e.g., “I have no difficulty concentrating”) and four items measure “absorption by activity” (e.g., “I don’t notice time passing”) on a 7-point scale (1 =“not at all” - 7 = “very much”). Additionally, 3 items measure perceived importance or perceived outcome importance (concern, e.g., “I must not make any mistakes here”) and 3 further items assess demand, skills, and the perceived fit of demands and skills (e.g., “Compared to all other activities which I partake in, this one is…”–answers are possible on a 9-point scale ranging from 1 =“easy” to 9 =“difficult”). The mean of each component was used as dependent variable in the analyses.

The User Experience Questionnaire (UEQ) is generally used to assess interaction quality of certain design variations of (software) products and whether these catch the user’s attention and interest [73]. Accordingly, this questionnaire assesses conventional usability aspects or (goal-directed) pragmatic quality (efficiency, transparency/perspicuity, controllability/dependability), user experience or (non-goal-directed) hedonic quality (novelty, stimulation), as well as attractiveness by using bipolar ratings, such as “unpleasant—pleasant” or “appealing–repelling”. The subscale *attractiveness* describes the overall impression of the task, whether participants like or dislike it. *Transparency/perspicuity* describes the easiness to get familiar with the task and to learn how to use it. *Controllability/dependability* is related to the users’ feeling of control of the interaction and whether it is secure and predictable. The subscale *efficiency* reflects whether the user can solve the tasks without unnecessary effort and whether it reacts fast. *Novelty* describes whether participants find the design of the task creative and whether it catches their interest. The subscale *stimulation* reflects whether the task is exciting and motivating and whether it is fun to use. Transformed values range from -3 to +3, where +3 represents the most positive and the -3 the most negative value [74].

To measure affective responses (mood and emotion), we used the German version of the Positive and Negative Affect Schedule (PANAS) [72]. The PANAS comprises 20 items, with 10 items measuring positive affect (e.g., excited, inspired) and 10 items measuring negative affect (e.g., upset, afraid). Each item is rated on a five-point scale, ranging from 1 = “Not at all” to 5 = “Extremely”, to measure the extent to which the respective affect has been experienced in a specified time frame. The items can be split up to four subscales: Joy, activity/interest, afraid, upset.

### Procedure

Participants were tested individually. After providing informed consent and reading the instructions, NIRS optodes were mounted on their heads and quality of the NIRS signals being recorded was checked. Then, half of the participants started with the game-based version and the other half started with the non-game-based version of the number line estimation task. Counterbalancing of the task versions was done in a pseudo-randomized order so that the same number of male and female participants started with the same task version. Before each task, one practice trial (1 level including 8 tasks) had to be performed. After completing the first version (6 levels, each including 8 tasks), participants completed the FKS, UEQ, and PANAS questionnaires to assess their subjective experience during the previously completed task. Then, the second version had to be performed before completing the FKS, UEQ, and PANAS questionnaires again. Overall, the experiment took about one hour.

### Statistical analysis

To identify NIRS channels that showed the strongest NIRS signal change (oxy- and deoxy-Hb) due to the game-based vs. non-game-based version (average of second 0 to 50 after level onset), we used the False Discovery Rate (FDR) method to control the proportion of false positives among the channels that are detected as significant [75]. Therefore, one sample *t*-tests against 0 were calculated per channel and version to reveal whether relative NIRS signal changes differ from 0. Significant differences from 0 in oxy- and deoxy-Hb were then determined by using the FDR method.

To compare NIRS signal change between the game-based and non-game-based task version statistically, analyses of variances (ANOVAs) for repeated measures were applied including the within-subject factors *TASK-VERSION* (game-based vs. non-game-based version) and *HEMISPHERE* (left vs. right), separately for oxy- and deoxy-Hb. In particular, separate ANOVAs for different regions of interest (ROIs) were performed. Therefore, the 22 NIRS channels were merged to 9 ROIs (ROI1: channels 1 & 2; ROI2: channels 3 & 4; ROI3: channels 5, 10, and 14; ROI4: channels 6 & 15; ROI5: channels 7, 11, 12, and 16; ROI6: channels 8 & 17; ROI7: channels 9, 13, and 18; ROI8: channels 19 & 20; ROI9: channels 21 & 22; see also Table 1 and Fig 2). One ANOVA was performed for ROIs over the left vs. right orbitofrontal cortex (ROI 1 vs. ROI 2), one ANOVA was performed for ROIs over the left vs. right middle and inferior frontal cortex (ROI 3 vs. ROI 7), one for ROIs over the left vs. right superior frontal cortex (ROI 4 vs. ROI 6), one for ROIs over the left vs. right middle/superior frontal cortex (ROI 8 vs. ROI 9), and one ANOVA with the within-subject factor *TASK-VERSION* over the medial superior frontal cortex (ROI 5).

To test performance differences in the number line estimation task, we ran paired *t*-tests on error rate, mean estimation accuracy, and mean duration per task comparing game-based and non-game-based version. To evaluate differences between game-based and non-game-based version in the questionnaire data, we also ran paired sample *t*-tests.

For statistical analyses, the probability of a Type I error was maintained at 0.05. For the paired *t*-tests and multiple ANOVAs, Bonferroni-Holm corrections for multiple comparisons were applied [76]. To detect outliers, data were z-transformed per version. Values larger or smaller +/- 3 SD were rejected from statistical analysis (7% of NIRS data).

## Results

### NIRS-signal: Topographical distribution

**Fig 3:**
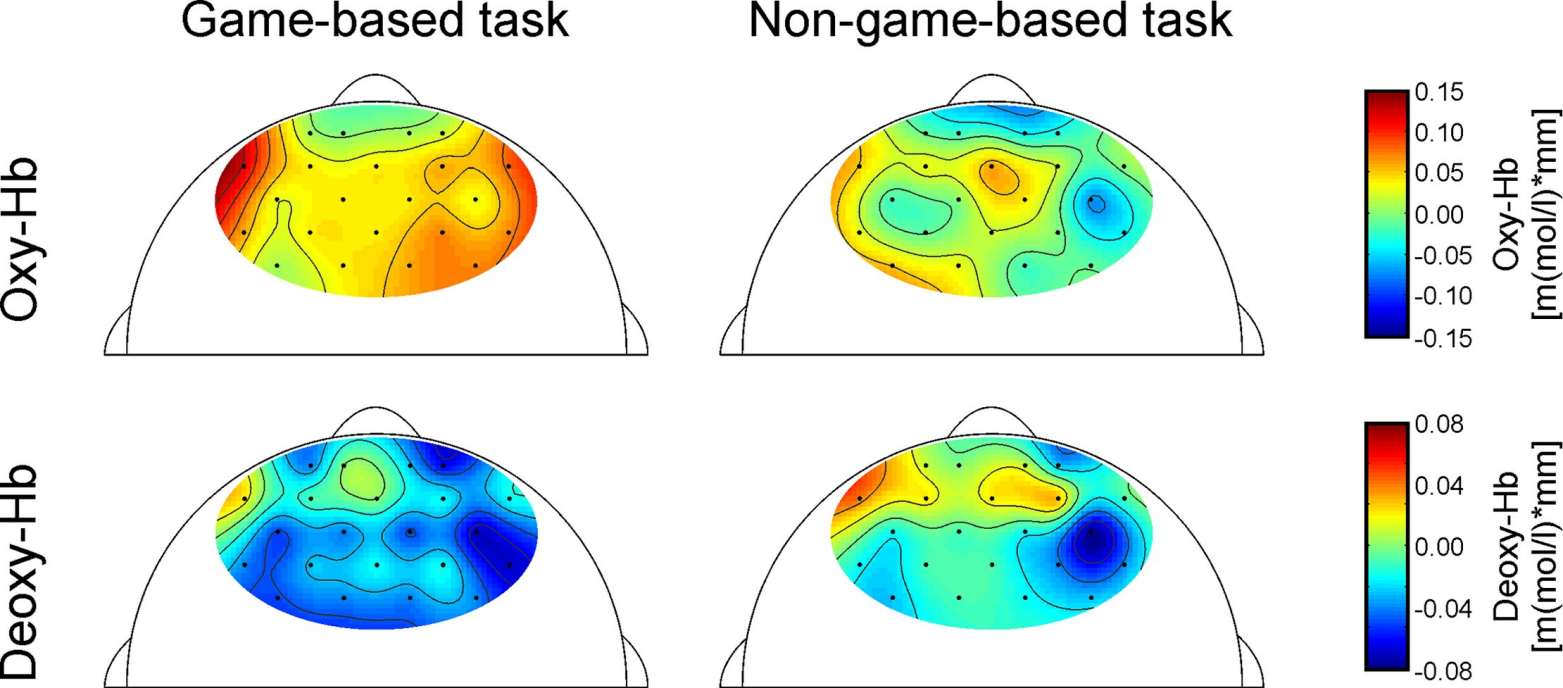
NIRS topography. Topographical distribution of oxy- and deoxy-Hb during the game-based and non-game-based version.

As illustrated in Table 2, oxy-Hb significantly increased over channels 5, 8, and 17 for the game-based version, whereas no significant increase or decrease could be observed for the non-game-based version. Deoxy-Hb significantly decreased over 15 of the recorded 22 channels in the game-based version. In the non- game-based version, deoxy-Hb significantly decreased over channel 13, but increased over channel 5. The topographical distribution of oxy- and deoxy-Hb per version is depicted in Fig 3.

**Table 2:** Results of topographical analysis after FDR correction.

Oxy-Hb Game-based task		Oxy-Hb Non-game-based task		Deoxy-Hb Game-based task		Deoxy-Hb Non-game-based task		
Ch. Nr.	*p*-value	Ch. Nr.	*p*-value	Ch. Nr.	*p*-value	Ch. Nr.	*p*-value	Critical *p*-value FDR
Ch5	8.98E-05*	Ch7	0.0289	Ch13	2.27E-05*	Ch13	1.38E-05*	0.0045
Ch17	0.0037*	Ch6	0.0529	Ch4	3.8E-05*	Ch5	0.0020*	0.0091
Ch8	0.0109*	Ch19	0.0673	Ch12	5.53E-05*	Ch17	0.0362	0.0136
Ch22	0.0221	Ch13	0.0757	Ch18	0.0001*	Ch18	0.0400	0.0182
Ch14	0.0227	Ch4	0.0899	Ch11	0.0006*	Ch8	0.0410	0.0227
Ch18	0.0254	Ch8	0.1160	Ch19	0.0006*	Ch4	0.0826	0.0273
Ch9	0.0268	Ch10	0.1347	Ch10	0.0007*	Ch14	0.0884	0.0318
Ch21	0.0480	Ch3	0.1765	Ch14	0.0008*	Ch22	0.1059	0.0364
Ch15	0.0700	Ch15	0.1950	Ch21	0.0013*	Ch19	0.1147	0.0409
Ch7	0.0734	Ch12	0.2103	Ch3	0.0025*	Ch6	0.1187	0.0455
Ch6	0.1037	Ch5	0.2982	Ch1	0.0033*	Ch7	0.1223	0.0500
Ch11	0.1152	Ch20	0.4103	Ch20	0.0044*	Ch12	0.1293	0.0545
Ch12	0.1280	Ch18	0.4513	Ch22	0.0085*	Ch21	0.1486	0.0591
Ch16	0.2110	Ch2	0.4587	Ch15	0.0136*	Ch10	0.2168	0.0636
Ch13	0.2147	Ch21	0.4714	Ch17	0.0247*	Ch15	0.2239	0.0682
Ch20	0.2354	Ch16	0.5124	Ch16	0.1215	Ch16	0.3460	0.0727
Ch10	0.2526	Ch22	0.7056	Ch8	0.1672	Ch9	0.4599	0.0773
Ch3	0.5623	Ch1	0.7432	Ch9	0.2064	Ch11	0.4793	0.0818
Ch4	0.6094	Ch14	0.8166	Ch6	0.3190	Ch1	0.5635	0.0864
Ch19	0.7074	Ch11	0.9463	Ch5	0.3276	Ch20	0.5974	0.0909
Ch1	0.7613	Ch17	0.9957	Ch2	0.8083	Ch3	0.6601	0.0955
Ch2	0.8987	Ch9	0.9968	Ch7	0.9686	Ch2	0.7786	0.1000

*p*-values of the one sample *t*-tests again 0 are listed in ascending order per channel and task version (game-based vs. non-game-based version), presented separately for oxy- and deoxy-Hb values. Additionally, critical *p*-values as determined by the FDR correction are shown. Significant results are marked with asterisks. Ch: Channel; FDR: False discovery rate.

### NIRS-signal: Game-based vs. non-game-based task

**Fig 4:**
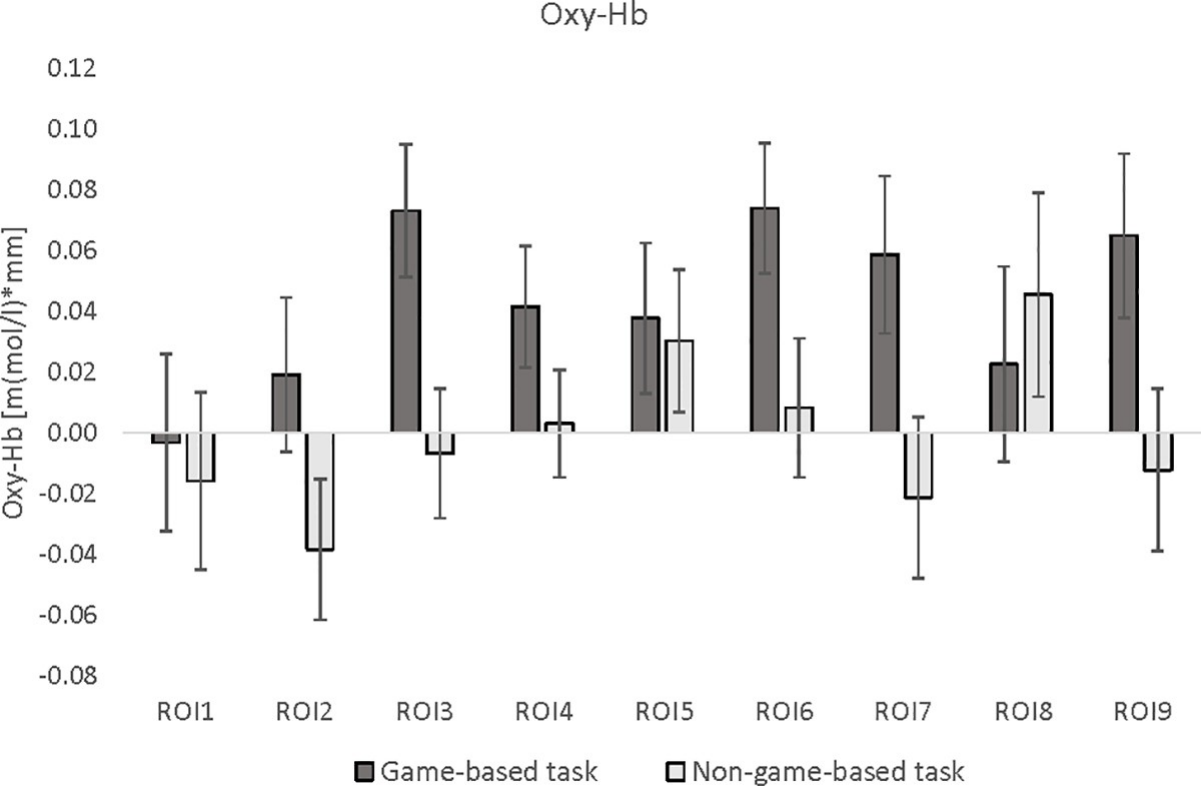
ANOVA results for oxy-Hb. Means and *SE* of oxy-Hb in the 9 regions of interests (ROI), presented separately for the game-based and non-game-based version. ROI 1: left orbitofrontal cortex; ROI 2: right orbitofrontal cortex; ROI 3: left middle/inferior frontal cortex; ROI 4: left superior frontal cortex; ROI 5: medial superior frontal cortex; ROI 6: right superior frontal cortex; ROI 7: right middle/inferior frontal cortex; ROI 8: left middle/superior frontal cortex; ROI 9: right middle/superior frontal cortex.

**Fig 5:**
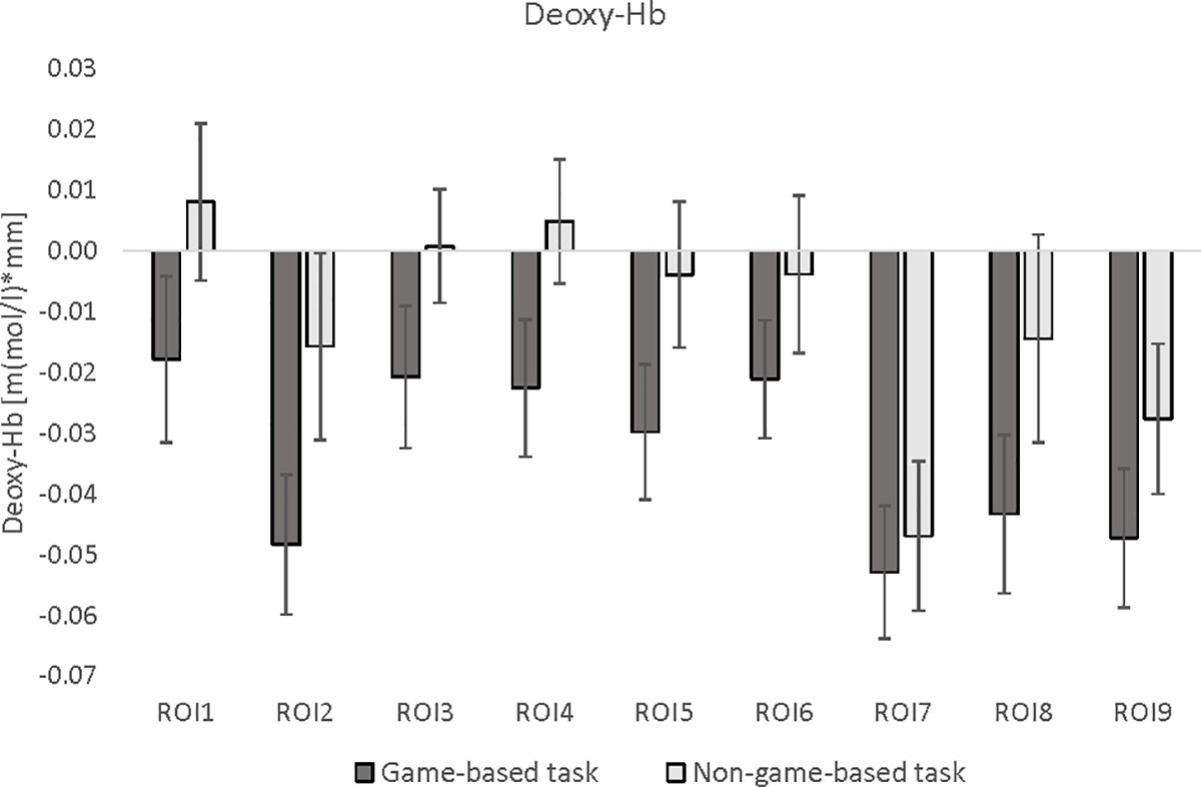
ANOVA results for deoxy-Hb. Means and *SE* of deoxy-Hb in the 9 regions of interests (ROI), presented separately for the game-based and non-game-based version. ROI 1: left orbitofrontal cortex; ROI 2: right orbitofrontal cortex; ROI 3: left middle/inferior frontal cortex; ROI 4: left superior frontal cortex; ROI 5: medial superior frontal cortex; ROI 6: right superior frontal cortex; ROI 7: right middle/inferior frontal cortex; ROI 8: left middle/superior frontal cortex; ROI 9: right middle/superior frontal cortex.

To analyze differences in brain activation patterns between the game-based and the non-game-based task version, different ANOVAs with the within-subject factors *TASK-VERSION* and *HEMISPHERE* (left vs. right) were calculated separately for oxy- and deoxy-Hb. Fig 4 illustrates mean oxy-Hb values for each ROI and task version. In Fig 5, mean deoxy-Hb values per ROI and task are shown.

Oxy-Hb orbitofrontal cortex (ROI 1 vs. ROI 2): The ANOVA revealed no significant results.

Oxy-Hb middle and inferior frontal cortex (ROI 3 vs. ROI 7): The main effect *TASK-VERSION* (*F*(1,58) = 10.90, *p*<0.01, *ɳ_p_^2^* = 0.18) was significant. Activation was stronger in the game-based than in the non-game-based version (Fig 4).

Oxy-Hb superior frontal cortex (ROI 4 vs. ROI 6): The main effect *TASK-VERSION* (*F*(1,58) = 8.51, *p*<0.01, *ɳ_p_^2^* = 0.14) was significant indicating that activation was stronger in the game-based than in the non-game-based version (Fig 4).

Oxy-Hb middle/superior frontal cortex (ROI 8 vs. ROI 9): The interaction effect TASK-VERSION*HEMISPHERE was significant (*F*(1,58) = 8.40, *p*<0.01, *ɳ_p_^2^* = 0.14). The game-based version led to a stronger oxy-Hb increase than the non-game-based version over the right hemisphere (ROI 9), whereas no differences were observed over the left hemisphere (ROI 8, Fig 4).

Oxy-Hb medial superior frontal cortex (ROI 5): The ANOVA revealed no significant results.

Deoxy-Hb orbitofrontal cortex (ROI 1 vs. ROI 2): The main effect *TASK-VERSION* (*F*(1,58) = 5.36, *p*<0.05, *ɳ_p_^2^* = 0.10) was significant. Deoxy-Hb showed a stronger decrease in the game-based than in the non-game-based version (Fig 5). However, this effect was not significant any more after Bonferroni-Holm correction. Additionally, the significant main effect *HEMISPHERE* (*F*(1,58) = 8.62, *p*<0.01, *ɳ_p_^2^* = 0.15) indicated that the deoxy-Hb decrease was stronger over the right (ROI 2) than over the left orbitofrontal cortex (ROI 1, Fig 5).

Deoxy-Hb middle and inferior frontal cortex (ROI 3 vs. ROI 7): The significant main effect *HEMISPHERE* (*F*(1,58) = 12.04, *p*<0.01, *ɳ_p_^2^* = 0.20) indicated that the deoxy-Hb decrease was stronger over the right (ROI 7) than over the left middle/inferior frontal cortex (ROI 3, Fig 5).

Deoxy-Hb superior frontal cortex (ROI 4 vs. ROI 6): The ANOVA revealed no significant results. The main effect *TASK-VERSION* (*F*(1,58) = 3.62, *p* = 0.06, *ɳ_p_^2^* = 0.07) only showed a trend that deoxy-Hb showed a stronger decrease during the game-based than during the non-game-based version (Fig 5).

Deoxy-Hb middle/superior frontal cortex (ROI 8 vs. ROI 9): The ANOVA revealed no significant results.

Deoxy-Hb medial superior frontal cortex (ROI 5): The decrease in deoxy-Hb was by trend (*F*(1,58) = 3.12, *p* = 0.08, *ɳ_p_^2^* = 0.06) more pronounced during the game-based than during the non-game-based version (Fig 5).

**Fig 6:**
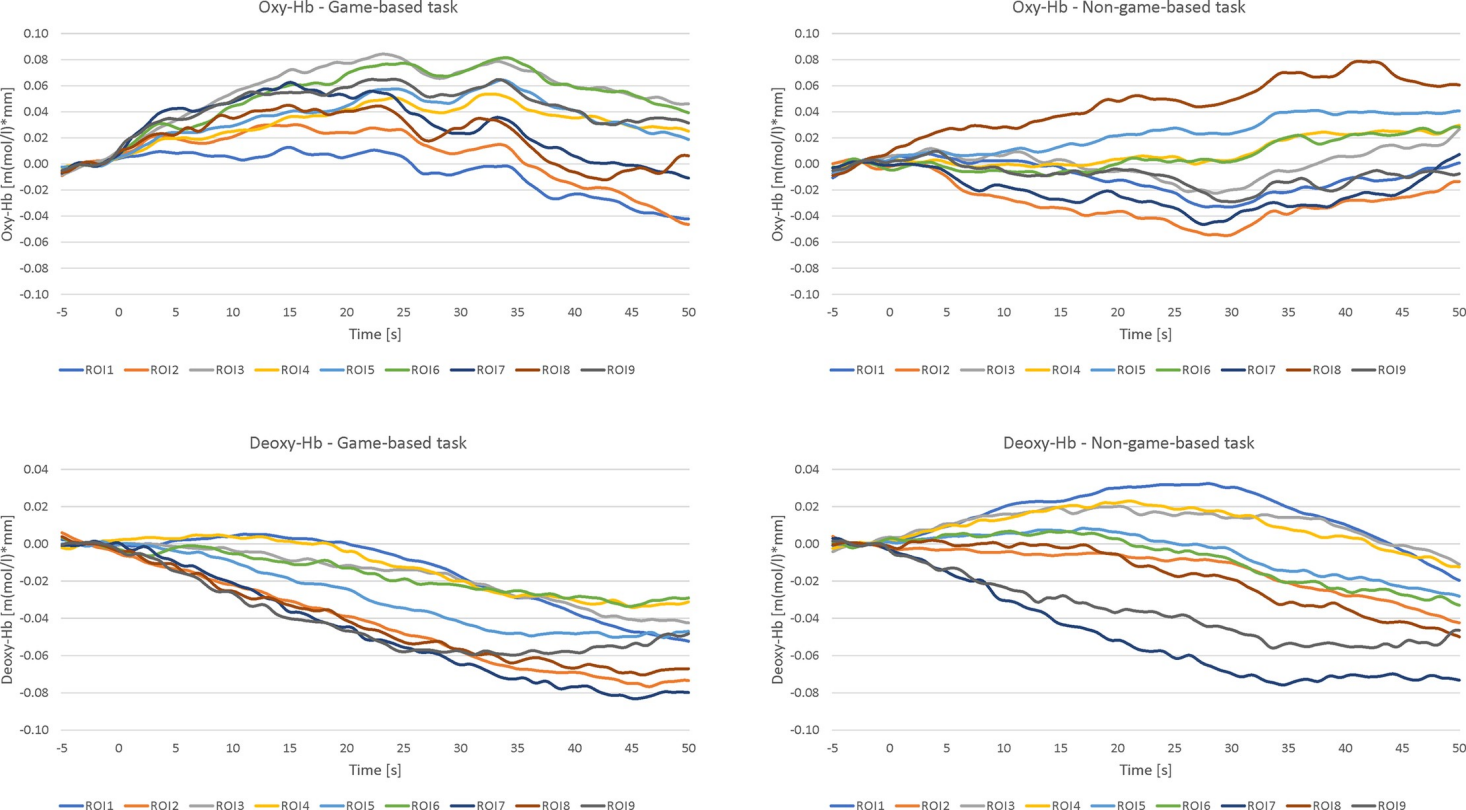
NIRS time courses. NIRS time course of oxy-Hb (upper panel) and deoxy-Hb (lower panel) during the game-based (left panel) and non-game-based (right panel) version, presented separately for each of the nine regions of interests (ROI). ROI 1: left orbitofrontal cortex; ROI 2: right orbitofrontal cortex; ROI 3: left middle/inferior frontal cortex; ROI 4: left superior frontal cortex; ROI 5: medial superior frontal cortex; ROI 6: right superior frontal cortex; ROI 7: right middle/inferior frontal cortex; ROI 8: left middle/superior frontal cortex; ROI 9: right middle/superior frontal cortex.

In Fig 6, the NIRS times courses of oxy- and deoxy-Hb are presented, separately for each of the 9 ROIs.

### Behavioral results and user experience

In Table 3, means and *SE* of behavioral and user experience data are summarized as well as results of the statistical comparison between the game-based and non-game-based task version.

**Table 3:** Behavioral and user experience data (means and *SE*) and results of the statistical comparison between the game-based and non-game-based task version.

		Game-based task	Non-game-based task	
		*Mean* (*SE*)	*Mean* (*SE*)	*p*-values of *t*-tests
**Estimation performance**	Error rate	24.98 (1.69)	28.76 (1.97)	0.02
	Mean Accuracy (%)	94.76 (0.20)	94.52 (0.21)	0.21
	Mean duration per task (ms)	8109.07 (264.44)	7930.53 (266.13)	0.49
**Flow**	Overall	4.50 (0.12)	4.43 (0.13)	0.49
	Fluency	4.72 (0.14)	4.70 (0.15)	0.84
	Absorption	4.18 (1.10)	4.04 (0.16)	0.35
	Perceived importance	2.24 (0.14)	1.96 (0.14)	0.04
	Perceived fit of demands and skills	4.18 (0.15)	3.99 (0.16)	0.05
**User experience**	Attractiveness	0.98 (0.12)	0.07 (0.15)	<0.001*
	Efficiency	1.04 (0.09)	1.30 (0.10)	0.007*
	Transparency/Perspicuity	1.95 (0.10)	2.12 (0.10)	0.11
	Controllability/Dependability	0.97 (0.11)	1.11 (0.10)	0.21
	Novelty	0.64 (0.15)	-0.78 (0.18)	<0.001*
	Stimulation	0.53 (0.16)	-0.09 (0.17)	0.002*
**Positive/ negative affect**	Joy	2.51 (0.09)	2.30 (0.09)	0.01*
	Activity/ Interest	3.35 (0.08)	3.25 (0.10)	0.24
	Afraid	1.29 (0.04)	1.26 (0.03)	0.32
	Upset	1.27 (0.38)	1.17 (0.29)	0.07

Significant results are marked with asterisks.

In the game-based version, less errors were made than in the non-game-based version (Table 3), although this difference was statistically no longer significant after Bonferroni-Holm correction. Accuracy and mean duration per task were comparable between versions. Flow experience did not differ between task versions (Table 3). There was only a non-significant trend that the perceived importance to be successful is higher in the game-based than in the non-game-based version. Additionally, the perceived fit of demands and skills seems to be descriptively higher in the game-based than in the non-game-based version. Results of the UEQ revealed that participants rated the game-based version to be more attractive, novel, and stimulating than the non-game-based version (Table 3). In contrast, participants experienced the non-game-based version as more efficient than the game-based version (Table 3). According to the results of the PANAS, participants experienced more joy during the game-based than during the non-game-based version (Table 3).

## Discussion

The primary aim of the present study was to investigate neuronal correlates of game-based learning by contrasting brain activation patterns while performing a game-based and a non-game-based version of a number line estimation task. We expected the game-based version to be more rewarding and emotionally engaging leading to a stronger activation in the prefrontal cortex. Possible differences in behavioral parameters such as task performance and user experience including flow and affect were examined in secondary and tertiary research questions, too. Since prior studies reported on heterogenous results concerning the effects of game-based learning tasks on user performance and experience, we did not formulate directed hypotheses in this context.

### Brain activation

Concerning our main research question, we found stronger activation of prefrontal brain areas when participants performed the game-based as compared to the non-game-based version. Generally, prior fMRI studies showed that a network of fronto-parietal brain areas seems to be involved in number line estimation including the superior and middle frontal gyrus and dorsolateral prefrontal cortex [51]. However, while the role of parietal areas in number processing is well established [i.e., subserving actual processing of number magnitude information; 77, 78], the role of frontal areas is less well understood and often considered to reflect also non-numerical mechanisms such as attention [for a meta-analysis see 79].

Prefrontal brain areas were also strongly activated in the present study, especially in the game-based version (relative increase in oxy-Hb and decrease in deoxy-Hb). Moreover, in a fMRI study by Vogel et al. (2013), number line estimation led to strong activation of bilateral prefrontal cortices, which was more pronounced for the right than on the left hemisphere in the superior and middle frontal gyrus. Results of the present study also indicate that activation patterns were bilaterally distributed, but in some ROIs, with stronger signal changes observed over the right hemisphere than over the left hemisphere. For instance, deoxy-Hb showed a stronger decrease over the right orbitofrontal cortex and middle and inferior frontal cortex than over the left orbitofrontal cortex and middle and inferior frontal cortex. However, analysis of activation changes over single channels revealed also strong activation patterns over the left hemisphere, such as a strong increase in oxy-Hb over the left inferior / middle frontal cortex. Generally, these results are well in line with the findings by Vogel et al. (2013). In summary, brain activation patterns observed are comparable with previous neuroimaging evidence on the neuronal correlates of number line estimation. These prefrontal brain activation patterns (increase in oxy- and decrease in deoxy-Hb) were more pronounced for the game-based as compared to the non-game-based version and might indicate that participants paid more attention in the game-based version [79].

Moreover, the game-based version might have been more rewarding than the non-game-based version leading to increased attention towards the more rewarding environment and associated stronger prefrontal activation in turn. Playing computer games was observed to be able to activate the reward system of the brain [6, 10, 34–38]. Neuroimaging studies reported a positive association between increased prefrontal brain activity and reward processing [36, 39–43]. For instance, the orbitofrontal cortex (together with anterior cingulate cortex, ACC) was argued to be involved in providing a motivational value of cue-inducing stimuli [6, 80]. Activation in the orbitofrontal cortex was found to decrease in the absence of an expected reward [6, 10]. The game elements included in the game-based version, such as the more pronounced positive feedback for successful performance (earning reward points, gestures of the game character, etc.), are assumed to be rewarding [7, 8]. This is backed by subjective ratings of the participants that also indicated the game-based version to be experienced as more rewarding than the non-game-based version. Hence, game elements might have led to stronger reward processing, which was associated with prefrontal brain activation, previously.

Another explanation for the stronger prefrontal activation in the game-based as compared to the non-game-based version might be that the emotional appeal was stronger in the game-based than in the non-game-based version [5]. In particular, we found increased activation (stronger decrease in deoxy-Hb) in the orbitofrontal cortex which is–amongst other areas–involved in emotion regulation and reliably activated by feelings of pleasure [for a review see 44]. In fact, recent studies indicated that game elements may well influence emotional states [5, 14, 81, 82]. Our neurofunctional results are in line with this idea, which is further corroborated by participants’ subjective ratings with positive affect (joy) rated higher in the game-based as compared to the non-game-based version. Generally, it was observed that emotional processing, whether positive or negative, lead to increased activation in prefrontal brain regions [83–85]. This indicates that the increased prefrontal activation in the game-based version might be caused by increased emotional processing or emotional engagement in the gaming task.

Moreover, previous neurofunctional studies found increased frontal brain activation during game-based task versions with higher flow experience [28, 32, 86]. Using NIRS, de Sampaio Barros et al. (2018) investigated neuronal correlates of flow experience during gaming. They found that oxy-Hb was highest in an optimal state of task demands experienced to balance player resources. In the present study, we also found higher levels of oxy-Hb in the game-based version, for which participants reported that their perceived fit of demands and skills was higher (although statistically not significant) than in the non-game-based version. However, as we did not find any statistically significant differences in the subjective flow experience between task versions, differences in flow experience may not explain the differences in prefrontal activation.

Differences in task difficulty or cognitive load [10, 29, 31, 33, 87–89] can also not explain differences in prefrontal activation patterns between the two task versions because task difficulty was comparable between the game-based and non-game-based version of the present study (with even a trend towards better performance in the game-based version as reflected in errors committed). However, although differences in game elements between the two versions did not lead to significant differences in behavioral performance, one cannot exclude that the need of processing the richer input (i.e., added game elements) in the game-based version led to increased frontal activation [90].

### Behavioral performance

Behavioral performance was comparable between the two task versions. Although participants made fewer errors in the game-based as compared to the non-game-based version, this difference in behavioral performance was statistically no longer significant after controlling for multiple comparisons. The observed comparable behavioral performance in both task versions shows on the one hand, that game elements did not interfere with participants’ task performance, as suggested by literature on seductive details [55–57]. In turn, this might indicate that game elements were integrated in a coherent and meaningful way and connected to the task [55, 91]. On the other hand, adding game elements to a learning task–here to the number line estimation task–did not lead to a statistically significant performance improvement as found in previous studies [for a meta-analysis see 4].

However, it has to be noted that the majority of previous studies used interventions, in which participants performed the game-based learning task over repeated and/or longer training sessions. In the present study, we acquired neurofunctional data and therefore participants completed only one session. Hence, one may not assume that fraction knowledge was fostered significantly within this single session. Moreover, the estimation accuracy of the participants was already high and thus clear improvements were not even expected. Taken together, our results indicate that adding game elements to a number line estimation task had neither significant beneficial nor negative effects on task performance within one session.

### User experience, affect, and flow

In contrast to behavioral performance, subjective user experience differed significantly between task versions. Participants rated the game-based version as more attractive, novel, and stimulating than the non-game-based version. Hence, they seemed to like the game-based version more than the non-game-based version (subscale attractiveness), experienced the game-based version as more exciting and motivating (subscale stimulation), and found the game-based version more creative, so that it caught their interest more than the non-game-based version (subscale novelty) [74]. Additionally, positive affect was higher during the game-based than the non-game-based task. This is in line with prior findings. For instance, Heidig et al. (2015) found that the perceived aesthetics of a game learning task positively affects the emotional states of the learners. While the emotional states had a minor impact on learning outcomes, they had a larger impact on intrinsic motivation, including motivation to continue working with the material [81]. Kiili, Lindstedt, and Ninaus (2018) also found that intrinsically motivated students showed higher positive affect when playing Semideus over a longer time period [61]. In a similar study, Ninaus et al. (2019) [14] found overall increased emotional engagement in a game-based compared to a non-game-based version of a number line estimation task utilizing both conventional paper-pencil questionnaires as well as automatic facial emotion detection. Hence, adding game elements to the task increased the attractiveness of the task and increased positive affect, which might positively affect motivation and the time learners spent with the learning environment. However, it has been suggested, that beneficial aspects of game elements require careful balancing of emotional features as these features might also overstrain individual’s emotional regulation capabilities [92, 93].

Furthermore, it needs to be mentioned that even though participants rated the game-based version as attractive, novel, and stimulating, they also rated the game-based version of the task as less efficient than the non-game-based version. However, this subjective user experience was not reflected by the behavioral performance because both task versions led to comparable performance in the number line estimation task.

We also assessed flow experience, because flow is one the most popular motivational constructs assessed in gaming studies and suggested to positively affect learning outcomes [58]. We did not find any statistically significant differences in the flow experience between the two task versions. There was only a non-significant trend that the perceived importance to be successful was higher in the game-based than in the non-game-based version. Additionally, the perceived fit of demands and skills was descriptively higher in the game-based as compared to the non-game-based version. In a similar study, Ninaus et al. (2019) observed higher levels of flow in a game-based version compared to a non-game-based version of a number line estimation task [14]. However, the authors used a larger sample and thus were able to identify even smaller effects. Kiili and Ketamo (2018) [59] also found increased flow experience in a game-based number line estimation task when compared to its paper based non-game-based equivalent. Accordingly, not only game elements but also the medium (digital vs. paper-based) differed between the two conditions, which makes a clear interpretation rather difficult. Future studies will need to further investigate the effects of game elements on flow experience.

Subjective flow experience is often associated with increased performance and learning gains, because flow constitutes a state of optimal balance between challenge and skill [60, 63, 64]. Probably, we did not find any differences in flow experience between task versions because of the lack of performance differences between the game-based and non-game-based version. That is, the game-based and non-game-based task versions were equally challenging. However, there are also prior studies that found significant performance differences between a game-based and a non-game-based task version but no differences in flow experience [9]. Interindividual differences in flow experience might also contribute to the non-significant results [94, 95].

### Limitations

Performing only one fraction estimation session (7 minutes on average per task) was too short to reveal any significant differences in learning effects. Future studies investigating changes in neuronal correlates when performing this learning task over a longer time period comparing a game-based and a non-game-based task version are necessary to evaluate differential learning effects.

Concerning the increased frontal brain activation in the game-based task version, we cannot exclude that the need of processing the richer input (i.e., added game elements) in the game-based version led to a higher cognitive or mental workload and consequently to increased frontal activation patterns as already discussed before [90]. However, we did not observe significant differences in behavioral performance between the two task versions, which one might expect in case of high cognitive or mental workload due to the inclusion of game elements.

## Conclusions and implications

The present study investigated neuronal correlates of adding game elements to a number line estimation task. We compared frontal activation patterns between a game-based and a non-game-based version of the same task. Additionally, possible differences in user performance and subjective user experience between task versions were investigated. Results showed that adding game elements to a learning task such as number line estimation lead to stronger activation of brain areas, which were previously observed to be involved in a non-game-based number line estimation task.

Within one session we observed that prefrontal brain areas were activated more strongly in the game-based version, including game elements, as compared to the non-game-based version. This might reflect higher demands on reward and emotional processing as well as stronger attention towards the learning material when game elements are added. Hence, game-based learning tasks might also lead to a superior learning outcome in future training studies. In particular, the association between neurofunctional activity in game-based learning and learning outcomes need to be investigated further.

In this context, adding game elements to traditional learning tasks used at school (e.g., verbal tasks, math tasks, natural science) might have beneficial effects, too. Additionally, game elements might be used to design learning tasks, which have to be performed at home. Recently, the COVID-19 epidemic caused a school lockdown and a switch to digital learning. Many students were forced to perform their learning tasks at home with remote supervision of their teachers. This turned out to be challenging for students, teachers, as well as parents [96, 97]. Here, using game-based learning environments might potentially improve home-based, digital learning outcomes and students’ motivation in the future. Summing up, results of the present study underscore the potential of game-based learning to promote more efficient learning by means of attention and reward up-tuning.
